# Analysis of Prosthetic Joint Infections Following Invasive Dental Procedures in England

**DOI:** 10.1001/jamanetworkopen.2021.42987

**Published:** 2022-01-19

**Authors:** Martin H. Thornhill, Annabel Crum, Saleema Rex, Tony Stone, Richard Campbell, Mike Bradburn, Veronica Fibisan, Peter B. Lockhart, Bryan Springer, Larry M. Baddour, Jon Nicholl

**Affiliations:** 1 Unit of Oral and Maxillofacial Medicine Surgery and Pathology, School of Clinical Dentistry, University of Sheffield, Sheffield, United Kingdom; 2 Department of Oral Medicine, Carolinas Medical Center, Atrium Health, Charlotte, North Carolina; 3 School of Health and Related Research, University of Sheffield, Sheffield, United Kingdom; 4 Joint Replacement Surgeon, OrthoCarolina, Charlotte, North Carolina; 5 Division of Infectious Diseases, Mayo Clinic College of Medicine, Rochester, Minnesota

## Abstract

**Importance:**

Dentists in the United States are under pressure from orthopedic surgeons and their patients with prosthetic joints to provide antibiotic prophylaxis before invasive dental procedures (IDP) to reduce the risk of late prosthetic joint infection (LPJI). This has been a common practice for decades, despite a lack of evidence for an association between IDP and LPJI, a lack of evidence of antibiotic prophylaxis efficacy, cost of providing antibiotic prophylaxis, and risk of both adverse drug reactions and the potential for promoting antibiotic resistance.

**Objective:**

To quantify any temporal association between IDP and subsequent LPJI.

**Design, Setting, and Participants:**

This cohort study used a case-crossover and time trend design to examine any potential association between IDP and LPJI. The population of England (55 million) was chosen because antibiotic prophylaxis has never been recommended to prevent LPJI in England, and any association between IDP and LPJI would therefore be fully exposed. All patients admitted to hospitals in England for LPJI from December 25, 2011, through March 31, 2017, and for whom dental records were available were included. Analyses were performed between May 2018 and June 2021.

**Exposures:**

Exposure to IDP.

**Main Outcomes and Measures:**

The main outcome was the incidence of IDP in the 3 months before LPJI hospital admission (case period) compared with the incidence in the 12 months before that (control period).

**Results:**

A total of 9427 LPJI hospital admissions with dental records (mean [SD] patient age, 67.8 [13.1] years) were identified, including 4897 (52.0%) men and 4529 (48.0%) women. Of these, 2385 (25.3%) had hip prosthetic joints, 3168 (33.6%) had knee prosthetic joints, 259 (2.8%) had other prosthetic joints, and 3615 (38.4%) had unknown prosthetic joint types. There was no significant temporal association between IDP and subsequent LPJI. Indeed, there was a lower incidence of IDP in the 3 months prior to LPJI (incidence rate ratio, 0.89; 95% CI, 0.82-0.96; *P* = .002).

**Conclusions and Relevance:**

These findings suggest that there is no rationale to administer antibiotic prophylaxis before IDP in patients with prosthetic joints.

## Key Points

**Question:**

Is there an association between invasive dental procedures (IDP) and late prosthetic joint infections (LPJI) in patients who did not receive antibiotic prophylaxis prior to IDP?

**Findings:**

This cohort study of 9427 LPJI hospital admissions for which dental records were available from 15 months preceding admission found no evidence of a temporal association between IDP and LPJI.

**Meaning:**

These findings refute recommendations to give antibiotic prophylaxis to patients with prosthetic joints prior to IDP, given the cost, adverse drug reaction risk, and potential for promoting antibiotic resistance associated with antibiotic prophylaxis.

## Teaser

This cohort assesses whether there is a temporal association between invasive dental procedures and subsequent later prosthetic joint infection among patients in England.

## Introduction

Replacing arthritic joints with prosthetic joints is one of the great advances of modern medicine, with 2.9 million joints replaced annually worldwide.^1,2^ Periprosthetic joint infection (PJI) is a leading cause of arthroplasty failure. Early infections, within 3 months of joint replacement, are considered the result of wound contamination at the time of the surgical procedure. Early infection rates in the 1950s were approximately 12%, but antibiotic prophylaxis before joint replacement and lamina airflow operating rooms have reduced this to 1% to 2%,^3^ and refocused attention on late PJIs (LPJIs), which occur 3 months or longer after joint replacement operations.

LPJI may result in prosthesis removal, or more rarely, in loss of limb or life.^4^ The cost of treating LPJI is 4- to 6-fold that of the original arthroplasty^5,6,7,8^ and is projected to have cost $1.62 billion in the US in 2020,^9^ without taking account of the personal and societal costs of long-term disability or the impact on patients’ quality of life.^10^ Therefore, LPJI is of significant concern for the 28 000 US orthopedic surgeons and more than 7 million individuals with prosthetic joints.^4,11^ Furthermore, the number of individuals with prosthetic joints is increasing rapidly, with approximately 4 million new hip and knee arthroplasties projected annually in the US by 2030.^12^ Although LPJI incidence is comparatively low, it remains the most common mode of failure of knee replacement and the second most common of hip replacement, and the incidence is projected to increase with the increasing number of arthroplasties being performed.^4,13,14^

LPJI is most often attributed to hematogenous seeding of bacteria from another anatomical site,^15,16^ and this led US orthopedic surgeons to recommend patients with prosthetic joints be given antibiotic prophylaxis prior to invasive dental procedures (IDP),^17,18,19^ a practice that is supported by more than 90% of US orthopedic surgeons.^20,21^ Nonetheless, there are little data to support a causal link between IDP and LPJI, and there has never been a randomized clinical trial of antibiotic prophylaxis efficacy in preventing LPJI, to our knowledge. Furthermore, microbiological studies suggest that oral streptococci are an uncommon cause of LPJI, accounting for less than 10% of infections.^4,22,23,24,25,26,27,28^ These reasons may explain why antibiotic prophylaxis use for patients with prosthetic joints is not advocated in many countries, including the United Kingdom.^28^ The annual cost of providing antibiotic prophylaxis in the US has been calculated at approximately $59 640 000,^11^ but this does not take into account the cost of adverse drug reactions caused by antibiotic prophylaxis^28,29,30^ or the risk that antibiotic prophylaxis could contribute to selection of antibiotic resistant bacteria.^28,31,32^

For antibiotic prophylaxis to be effective, there must be a positive temporal association between IDP and LPJI, but data on this are lacking.^25^ In the absence of this evidence, the demand on US dentists to provide antibiotic prophylaxis for IDP remains controversial. The aim of this study was to determine whether there is a positive association between LPJI and IDP in the population of England where, because antibiotic prophylaxis is not recommended, the association should be fully exposed.

## Methods

This cohort study used national data and necessitated the transfer of individually identifying National Health Service (NHS) information between NHS Digital and the NHS Business Services Authority (NHSBSA); therefore, we obtained national research ethics approval and Confidentiality Advisory Group approval to process patient identifiable information without consent from the NHS Health Research Authority. We also obtained approval from NHS Digital’s Data Access Request Service and the Independent Group Advising on the Release of Data. This study is reported following the Strengthening the Reporting of Observational Studies in Epidemiology (STROBE) reporting guideline.

### Data Source

All hospital admissions in England are recorded in the Hospital Episode Statistics (HES) database of NHS Digital. This database was searched to identify all patients admitted between December 25, 2011, and March 31, 2017, with an *International Statistical Classification of Diseases and Related Health Problems, Tenth Revision* (*ICD-10*)^33^ code for infection and inflammatory reaction due to internal joint prosthesis (T84.5) in any discharge diagnosis code field. From this list, NHS Digital created two data sets. The first data set was a full set of identifying patient details (NHS number, surname, forenames, date of birth, gender, full address, postcode). These identifiers, along with a unique study ID for each patient (encrypted HES ID), but no clinical information, were transferred to NHSBSA. The second data set was a full set of clinical, diagnostic, and procedural data for the period of January 1, 2000, through March 31, 2017, for all patients identified with *ICD-10* code T84.5, as well as data relating to the PJI admission. This allowed us to identify the time between joint replacement and PJI, and the type of joint replaced (eg, hip, knee). Data set 2 also included the same encrypted HES ID field included in data set 1, but no other patient identifying information.

NHSBSA maintains dental records for all patients receiving NHS dental treatment in England. Using the personal identifiers received from NHS Digital in data set 1, NHSBSA created data set 3, including dental records for each individual from October 1, 2010, through March 31, 2017. All patient identifiers (except common encrypted HES ID) were removed from this data set before transfer to the study team. Thus, we were able to link medical (data set 2) and dental (data set 3) records of each patient using the common encrypted HES ID field.

### IDP

Since April 2008, dentists working in the English NHS have been required to record if a patient had a dental extraction, scaling, or endodontic procedure as part of dental treatment. There is wide acceptance that these can result in bacteremia and were considered IDP for this study.^34,35^ As a control group, we identified courses of treatment restricted to a simple dental examination, with or without radiographs, that did not involve an IDP. Other courses of treatment were considered intermediate.

Previous studies have shown that more than 90% of distant site infections associated with IDP occur within 3 months, and this period is used widely to define distant site infections associated with IDP.^4,36,37,38,39,40,41,42^ Hence, we chose a 3-month window of risk to assess for a potential association between IDP and LPJI.

NHSBSA data provided start and end dates for each course of dental treatment. Rarely, treatment courses were not completed, such as owing to death or change of residence. Although such situations were rare, when they occurred, the clinical record was not always complete; therefore, for our main analysis, we used end date to define a course of treatment, thereby excluding any incomplete treatment.

### LPJI Hospital Admissions

A cohort of individuals who had an LPJI between December 25, 2011, and March 31, 2017, were identified using *ICD-10* code T84.5. By reviewing each patient’s HES record back to 2000, we identified the date and joint replaced using OPCS Classification of Interventions and Procedures version 4 (OPCS-4) joint replacement codes (eTable 1 in the Supplement). This allowed us to subanalyze data by joint replaced. Joint replacements were divided into all, hip (codes W37-W39, W46-W48), knee (codes W40-W42), other (codes W43-W45, W49-W51), and unknown. Unknown included joint replacements performed before 2000, for which no replacement code data were available to us, or when more than 1 type of joint was replaced (eg, hip and knee joints) and we did not know which had been infected. To ensure only patients with LPJI were analyzed, this information was also used to exclude patients admitted for PJI within 3 months of joint replacement. We also excluded any admission for PJI that occurred within 12 months of an earlier PJI admission as representing a reoccurrence of the same infection. In addition to the T84.5 code, coders can record supplementary codes to indicate the nature of the causal organism, and these were noted when recorded (eTable 2 in the Supplement).

### Case-Crossover Design

Maclure^43^ proposed the case-crossover method for studying the association of transient events with subsequent outcomes while eliminating control selection bias and confounding by constant within-participant characteristics. In case-crossover studies, each individual serves as their own control.

This study examined individuals with LPJI as the outcome and evaluated exposure to IDP. We compared the incidence of IDP in a predefined 3-month case period immediately before LPJI hospital admission, with incidence of IDP in the preceding 12-month control period (months 4-15).^43,44,45^ To confirm the timing of events, the monthly incidence of dental procedures over the 15-month period before LPJI hospital admission was plotted. We also performed a post hoc sensitivity analysis in which we compared the effect of using a 3-, 4- or 5-month case period (eFigure 1 in the Supplement). Some case-crossover studies have compared case periods with 1 or several control periods of the same duration. However, Mittleman et al^46^ have shown that sampling the control exposure frequency over a full year was twice as efficient as sampling control-periods equal in duration to the case-period, even when many such control periods were sampled.

### Statistical Analysis

The incidence of IDP was analyzed using a longitudinal negative binomial regression model in which the number of IDP was the dependent variable, the covariates were time period and admission number, and the duration of each period was the offset term. The primary contrast was the number of IDP in the 3 months prior to LPJI compared with the incidence of IDP over the previous 12 months. The type of IDP (ie, scaling, extraction, or endodontic), and the incidence of intermediate and noninvasive procedures were assessed using analogous models. Additional analyses were undertaken defined by the site of prosthesis (ie, hip, knee, other, or unknown).

Power calculations for self-controlled case series are given by Musonda.^47,48^ With a sample size of 9427 patients with LPJI with linked dental records, and assuming no association between IDP and LPJI, there was >90% power to detect a relative incidence of 1.09, ie, a 9% higher incidence of IDP, in the 3-month risk period compared with the matched control period.

All analyses were conducted using Stata statistical software version 16.1 (StataCorp). *P* values were 2-sided, and statistical significance was set at *P* < .05. Data were analyzed from May 2018 to June 2021.

## Results

### Population Characteristics

Of 23 133 patients admitted to hospitals with LPJI between December 25, 2011, and March 31, 2017, 9427 patients (40.8%) had dental records available and were included in analyses. The mean (SD) age of patients with dental records was 67.8 (13.1) years, 4897 (52.0%) were men and 4529 (48.0%) were women. The joints involved were hip (2385 patients [25.3%]), knee (3168 patients [33.6%]), other (259 patients [2.8%]), and unknown (3615 patients [38.4%]). The demographic characteristics of patients with LPJI with linked dental data were similar to all patients with LPJI overall (Table 1). In-hospital mortality associated with LPJI admission was 847 patients (3.7%) among all patients with LPJI and 211 patients (2.2%) among those with dental records. Causal organism data were recorded for 4338 patients (46.0%) for whom dental data were available. Within this group, 2314 LPJIs (53.3%) were recorded as caused by staphylococci, 408 LPJIs (9.4%) by oral streptococci, 213 LPJIs (4.9%) by other streptococci, 863 LPJIs (19.9%) by other organisms, and 540 LPJIs (12.5%) were recorded as mixed infection.

**Table 1.** Characteristics of the Study Population

Characteristics	LPJI hospital admissions, No. (%)	
	With linked dental data (n = 9427)	All (N = 23 133)
Age, y		
Mean (SD)	67.8 (13.1)	69.4 (13.7)
Median (range)	69 (3-101)	71 (0-103)
Gender		
Men	4897 (52.0)	11 697 (50.6)
Women	4529 (48.0)	11 429 (49.4)
Prosthetic joint type		
Any	9427	23 133
Hip	2385 (25.3)	5861 (25.3)
Knee	3168 (33.6)	7349 (31.8)
Other	259 (2.8)	524 (2.3)
Unknown^a^	3615 (38.4)	9399 (40.6)
Causal organism recorded		
No	5089 (54.0)	12 022 (52.0)
Yes	4338 (46.0)	11 111 (48.0)
Oral streptococci^b^	408 (9.4)	986 (8.9)
Other streptococci^b^	213 (4.9)	560 (5.0)
Staphylococci^b^	2314 (53.3)	5658 (50.9)
Other causal organisms^b^	863 (19.9)	2355 (21.2)
Mixed^b^	540 (12.5)	1552 (14.0)
Hospital admission details		
All admissions	9427 (100.0)	23 133 (100.0)
Elective^c^	5676 (60.2)	12 973 (56.1)
Emergency	3.751 (39.8)	10 160 (43.9)
Length of stay, d		
Mean (SD)	17.3 (25.1)	20.1 (28.3)
Median (range)	9 (0-389)	11 (0-646)
Discharged alive		
No	211 (2.2)	847 (3.7)
Yes	9191 (97.5)	22 164 (95.8)
Dental treatment details		
Course duration, d		
Mean (SD)	11.0 (31.0)	10.6 (31.0)
Median (range)	0 (0-854)	0 (0-1486)
All procedures	19 390 (100)	255 437 (100)
Invasive procedures	8930 (46.1)	113 058 (44.3)
Extractions^d^	1850 (20.7)	23 864 (21.1)
Scaling^d^	7313 (81.9)	92 102 (81.5)
Endodontic procedures^d^	308 (3.5)	3891 (3.4)
Intermediate procedures	2102 (10.8)	28 370 (11.1)
Non-invasive procedures	8358 (43.1)	114 009 (44.6)

Abbreviation: LPJI, late prosthetic joint infection.

^a^ Unknown includes joints inserted before 2000, when records started, and patients with multiple joints of different types for whom information on which joint was infected was not available.

^b^ Percentages shown are of LPJIs for which a causal organism was recorded.

^c^ Elective admissions include waiting list, booked, planned nonemergency transfers from another hospital, and admission method undefined.

^d^ Percentages shown are of invasive dental procedures. Numbers of dental procedures are the number of courses of dental treatment containing at least 1 of that type of procedure (the actual number of procedures could be more).

### Incidence of Different Dental Procedures During 15 Months Before LPJI Admission

**Figure 1.**
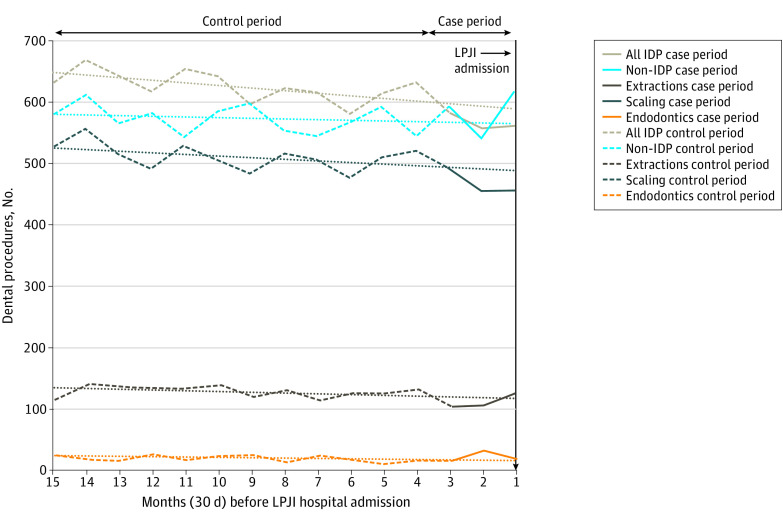
Monthly Incidence of Different Types of Dental Procedure During 15 Months Before Late Prosthetic Joint Infection (LPJI) Hospital Admission Dotted lines show the linear trend for the case-crossover study control period (months 4-15 before LPJI admission, dashed lines of the same color). The control period trend lines have been extended through the case period (months 1-3) to allow comparison between the control period trend and any changes in the incidence of dental procedures in the case period. IDP indicates invasive dental procedure.

Among 9427 patients admitted to hospitals with LPJI with dental records, the monthly incidence of IDP and noninvasive dental procedures in the 15 months before LPJI hospital admission were similar (Figure 1). In both groups, there was no significant increase in IDP in the case period (months 1-3 before LPJI admission) compared with the control period. Indeed, there was a significant decrease in IDP in the 3 months before LPJI admission (incidence rate ratio [IRR], 0.89; 95% CI, 0.82-0.96; *P* = .002, Table 2), although the significance of this decrease was lost when individual types of IDP were examined (scaling: IRR, 0.88; 95% CI, 0.79-1.01; *P* = .07; extractions: IRR, 0.84; 95% CI, 0.69-1.04; *P* = .11; endodontics: IRR, 1.15; 95% CI, 0.86-1.52; *P* = .34) and pairwise differences between different IDP types were not statistically significant. Post hoc sensitivity analyses using 4- or 5-month case periods also found no significant increase (or decrease) in IDP compared with control periods (eFigure 1 in the Supplement).

**Table 2.** Case-Crossover Analysis of LPJI Admissions With Linked Dental Data Comparing the Incidence of Dental Procedures in the 3-Month Case Period and the Preceding 12-Month Control Period

Procedure	LPJI															
	All (N = 9427)				Hip (n = 2385)				Knee (n = 3168)				Other/unknown (n = 3874)			
	Procedure, period		IRR (95% CI)	*P* value	Procedure, period		IRR (95% CI)	*P* value	Procedure, period		IRR (95% CI)	*P* value	Procedure, period		IRR (95% CI)	*P* value
	Case	Control			In Case	Control			Case	Control			Case	Control		
**Type of dental procedure**																
Invasive	568	627	0.89 (0.82-0.96)	.002	153	156	0.91 (0.80-1.04)	.18	197	214	0.90 (0.80-1.01)	.07	219	258	0.81 (0.69-0.96)	.01
Intermediate	129	147	0.74 (0.59-0.96)	.01	29	38	0.82 (0.64-1.04)	.11	43	49	0.90 (0.73-1.11)	.33	56	60	0.96 (0.80-1.14)	.62
Noninvasive	585	572	1.06 (0.98-1.14)	.14	147	144	1.07 (0.92-1.24)	.40	198	195	1.13 (1.01-1.26)	.03	240	234	0.99 (0.85-1.14)	.86
**Type of invasive dental procedure**																
Scaling	468	512	0.88 (0.79-1.01)	.07	132	129	1.01 (0.91-1.12)	.82	160	169	0.97 (0.89-1.06)	.52	177	214	0.85 (0.78-0.92)	<.001
Extractions	115	131	0.84 (0.69-1.04)	.11	29	31	0.93 (0.72-1.20)	.60	42	50	0.86 (0.71-1.05)	.15	44	50	0.91 (0.75-1.10)	.32
Endodontic	23	21	1.15 (0.86-1.52)	.34	4	4	1.11 (0.56-2.17)	.77	8	8	0.95 (0.59-1.53)	.83	11	9	1.35 (0.90-2.05)	.15

Abbreviations: IRR, incidence rate ratio; LPJI, late prosthetic joint infection.

### Site of Joint Replacement and LPJI

**Figure 2.**
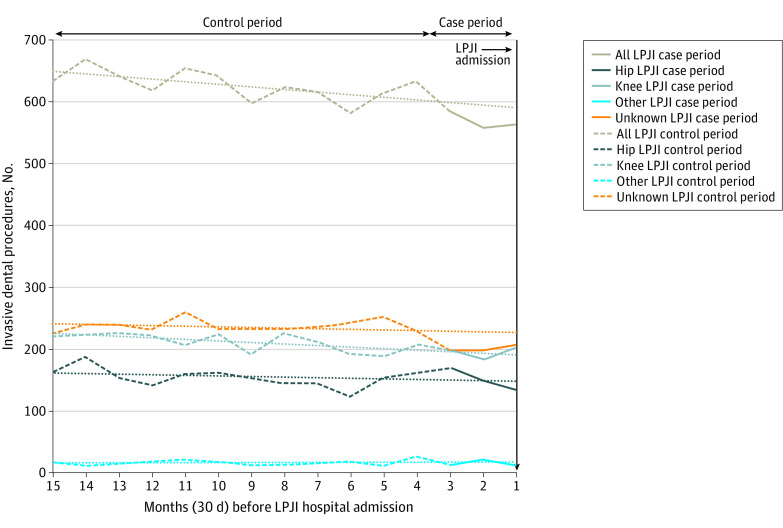
Monthly Incidence of Invasive Dental Procedures During 15 Months Before Admission to Hospital With Late Prosthetic Joint Infection (LPJI) Dotted lines show the linear trend for the case-crossover study control period (months 4-15 before LPJI admission, dashed lines of the same color). The control period trend lines have been extended through the case period (months 1-3, solid lines of the same color) to allow comparison between the control period trend and any changes in the incidence of dental procedures in the case period.

**Figure 3.**
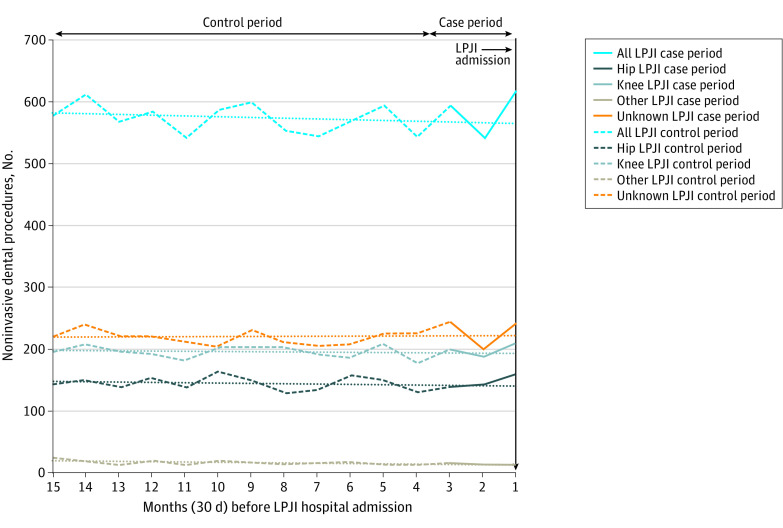
Monthly Incidence of Noninvasive Dental Procedures During 15 Months Before Admission to Hospital With Late Prosthetic Joint Infection (LPJI) Dotted lines show the linear trend for the case-crossover study control period (months 4-15 before LPJI admission, dashed lines of the same color). The control period trend lines have been extended through the case-period (months 1-3, solid lines of the same color) to allow comparison between the control period trend and any changes in the incidence of dental procedures in the case period.

Data were available on the site of the LPJI for 5812 patients (61.6%) with dental records (Table 1). Most LPJIs were in knee replacements (3168 patients [33.6%]), followed by hip replacements (2385 patients [25.3%]) and other joint replacements (259 patients [2.8%]). The site of joint replacement was not associated with IDP and LPJI, with no significant increase in IDP in the 3 months before LPJI admission, and pairwise differences between sites were not statistically significant (Table 2, Figure 2, and Figure 3; eFigures 2-5 in the Supplement).

## Discussion

In this cohort study of 9427 patients with dental records who developed LPJI, time trend and case-crossover analyses found no evidence of a temporal association between IDP and LPJI. In the 1970s and 1980s, the use of antibiotic prophylaxis to prevent infective endocarditis in individuals at high risk of LPJI undergoing IDP became well established and led US orthopedic surgeons to call for dentists to give antibiotic prophylaxis to patients with prosthetic joints.^17,18,19,49^ In 1988, the American Dental Association (ADA) sponsored a workshop to address this. Although there was limited evidence to support the use of antibiotic prophylaxis, they recommended its use until further evidence became available,^50,51^ and antibiotic prophylaxis was widely adopted by dentists.^52^ In 1997^53^ and 2003^54^ the ADA and American Academy of Orthopaedic Surgeons (AAOS) jointly published advisory statements recommending antibiotic prophylaxis for just 2 years after joint replacement but lifelong for patients with medical conditions that might put them at increased LPJI risk. However, in 2009, the AAOS unilaterally recommended that clinicians consider antibiotic prophylaxis for all patients with joint replacements prior to any invasive procedure that may cause bacteremia.^55^ This caused confusion among dentists and their patients.^11^ Several subsequent attempts were made by AAOS and ADA, either together or alone, to produce guidance,^56,57,58^ but these efforts only increased uncertainty about whether to provide antibiotic prophylaxis or not.^59,60,61^ As a result, in 2014, the ADA’s Council on Scientific Affairs assembled an expert panel to conduct a systematic review.^61^ They recommended: “In general, for patients with prosthetic joint implants, prophylactic antibiotics are not recommended prior to dental procedures.” Unfortunately, this advice lacked AAOS support. Consequently, there is still confusion among dentists and their patients, as well as ongoing pressure from orthopedic surgeons for their patients to receive antibiotic prophylaxis when undergoing IDP, and many dentists continue to give antibiotic prophylaxis for fear of being considered negligent.

There is little microbiological data to support a causal link between IDP and LPJI, and there has never been a randomized clinical trial of antibiotic prophylaxis to determine its safety and effectiveness in this context, to our knowledge. Unlike infective endocarditis, in which approximately 45% of infections are caused by oral streptococci, previous estimates suggest that oral streptococci are involved in less than 10% of LPJIs.^4,22,23,24,25,26,27,28^ Our study identified oral streptococci as a possible cause in approximately 9% of LPJIs. Nonetheless, we identified no increase in IDP prior to LPJI; if anything, there was a decrease. This suggests that those few LPJIs caused by oral streptococci were more likely a result of daily oral activities, such as toothbrushing, flossing, and mastication, particularly in patients with poor oral hygiene, rather than from IDP.^62^

For antibiotic prophylaxis to be effective, a positive causal link must exist between IDP and LPJI, and data on this are lacking.^25^ Only 5 studies have evaluated a potential association. In 1977, Waldman et al^63^ performed a retrospective case review of 62 patients who experienced late periprosthetic knee joint infection and identified 7 patients (11%) whose infections were temporally associated with IDP. In a related study, LaPorte et al^24^ temporally associated 3 of 52 (6%) late periprosthetic hip joint infections with IDP. However, neither study included a control group, making it impossible to draw any conclusions about the association between IDP and LPJI. In contrast, a case-control study by Kaandorp et al^37^ found that among 37 patients with LPJI, none had undergone an IDP in the previous 3 months, but 10% of control patients had. In a similar study of 42 Medicare patients with LPJI by Skaar et al,^40^ only 4 patients (9.5%) had undergone an IDP in the previous 3 months, compared to 15.9% of control patients. However, differences were not statistically significant in either study. In the largest study, by Berbari et al,^64^ 48% of 303 patients with PJI had undergone an IDP in the previous 2 years compared with 34% of 318 control patients, but a high proportion had received antibiotic prophylaxis. A subanalysis of patients who had not had antibiotic prophylaxis found 33 patients with PJI (11%) had an IDP in the previous 2 years, compared with 49 control patients (14%). None of the differences were statistically significant, and each study was hindered by small sample sizes and lack of statistical power. The case-control studies also suffered from selection bias and risk factor confounding between cases and controls. Furthermore, there was confounding due to the widespread use of antibiotic prophylaxis in the populations studied, and recall bias for dental procedure data was a problem in some studies.

This study is 30-fold larger than any previous study, to our knowledge, involving 9427 LPJI episodes for which dental records were available, and power calculations show the study had 90% power to detect a 9% difference in dental procedure incidence between case and control periods, which would be more than sufficient to identify any clinically significant association between IDP and LPJI. Furthermore, the confounding caused by antibiotic prophylaxis use in previously investigated populations was avoided by using the English population, where the use of antibiotic prophylaxis to prevent LPJI has never been advocated.^28^ Recall bias was eliminated by using NHS records of all events and their timing, and a major advantage of the case-crossover design is the avoidance of selection bias. This study design also implicitly accounts for many potential confounders (eg, differences in oral hygiene, comorbidities), since each individual serves as their own control.^43,44^

### Limitations

This study has several limitations. The T84.5 *ICD-10* code identifies PJI but does not identify the joint infected or distinguish between early and late PJI. To determine this, we searched each patient’s record for earlier joint replacement admissions to exclude early PJI (within 3 months of joint replacement). OPCS-4 joint replacement codes allowed us to identify the type of joint replaced, and this was used to subdivide episodes. However, because we could only access records from January 2000, if joint replacement occurred before that, we did not know the joint type replaced and had to record it as unknown.

We used supplementary *ICD-10* diagnosis codes to identify the LPJI causal organism. However, there are no *ICD-10* codes for oral viridans group streptococci, and we could only estimate their involvement by excluding other streptococcal species for which codes exist and assuming that any other or unspecified streptococci were oral. Therefore, it is likely that the 9% of LPJI assigned an oral streptococcal cause is an overestimate.

## Conclusions

The findings of this cohort study suggest that, in the absence of any increase in IDP prior to LPJI, there is no evidence to support a positive association between IDP and LPJI or the practice of administering antibiotic prophylaxis to patients with prosthetic joints undergoing IDP. The continuing use of antibiotic prophylaxis represents a large and unnecessary financial burden on individuals and the health care system as well as an unnecessary risk to patients, from adverse drug reactions, and society, owing to the potential development of antibiotic resistant bacteria, and should cease. However, our data suggest that maintenance of good oral hygiene may be important in preventing the small number of LPJIs in which oral bacteria are implicated. In addition, our findings should provide reassurance to orthopedic surgeons, dentists, and their patients in countries where antibiotic prophylaxis use in patients with prosthetic joints is not currently recommended.

## Notes

Supplement. **eTable 1.** OPCS-4 Procedure Codes for Joint Replacements **eTable 2.** Supplementary *ICD-10* Codes to Identify Whether a Causal Organism Was Recorded and the Nature of That Causal Organism **eFigure 1.** Sensitivity Analysis Using 3-Month, 4-Month and 5-Month Exposure Windows to IDP Before LPJI Admission (Case-Periods) and Different Control Periods in the Case-Crossover Analyses **eFigure 2.** Monthly Incidence of Different Dental Procedures Before Hip LPJI Admissions **eFigure 3.** Monthly Incidence of Different Dental Procedures Before Knee LPJI Admissions **eFigure 4.** Monthly Incidence of Different Dental Procedures Before Other LPJI Admissions **eFigure 5.** Monthly Incidence of Different Dental Procedures Before Unknown LPJI Admissions
