# Supplementary material for: Analysis of Prosthetic Joint Infections Following Invasive Dental Procedures in England
Source: JAMA Netw Open. 2022 Jan 19;5(1):e2142987. doi: 10.1001/jamanetworkopen.2021.42987 (PMC8771300; doi:10.1001/jamanetworkopen.2021.42987)
Supplement: Supplement. — eTable 1. OPCS-4 Procedure Codes for Joint Replacements eTable 2. Supplementary ICD-10 Codes to Identify Whether a Causal Organism Was Recorded and the Nature of That Causal Organism eFigure 1. Sensitivity Analysis Using 3-Month, 4-Month and 5-Month Exposure Windows to IDP Before LPJI Admission (Case-Periods) and Different Control Periods in the Case-Crossover Analyses eFigure 2. Monthly Incidence of Different Dental Procedures Before Hip LPJI Admissions eFigure 3. Monthly Incidence of Different Dental Procedures Before Knee LPJI Admissions eFigure 4. Monthly Incidence of Different Dental Procedures Before Other LPJI Admissions eFigure 5. Monthly Incidence of Different Dental Procedures Before Unknown LPJI Admissions [file jamanetwopen-e2142987-s001.pdf]

Supplemental Online Content

Thornhill MH, Crum A, Rex S, et al. Analysis of prosthetic joint infections following invasive dental procedures in England. *JAMA Netw Open*. 2022;5(1):e2142987.
doi:10.1001/jamanetworkopen.2021.42987

eTable 1. OPCS-4 Procedure Codes for Joint Replacements

eTable 2. Supplementary *ICD-10* Codes to Identify Whether a Causal Organism Was Recorded and the Nature of That Causal Organism

eFigure 1. Sensitivity Analysis Using 3-Month, 4-Month and 5-Month Exposure Windows to IDP Before LPJI Admission (Case-Periods) and Different Control Periods in the Case-Crossover Analyses

eFigure 2. Monthly Incidence of Different Dental Procedures Before Hip LPJI Admissions

eFigure 3. Monthly Incidence of Different Dental Procedures Before Knee LPJI Admissions

eFigure 4. Monthly Incidence of Different Dental Procedures Before Other LPJI Admissions

eFigure 5. Monthly Incidence of Different Dental Procedures Before Unknown LPJI Admissions

This supplemental material has been provided by the authors to give readers additional information about their work.

© 2022 Thornhill MH et al. *JAMA Network Open.*

eTable 1. OPCS-4 Procedure Codes for Joint Replacements

**Code**	**Procedure**
W37	Total prosthetic replacement of hip joint using cement
W38	Total prosthetic replacement of hip joint not using cement
W39	Other total prosthetic replacement of hip joint
W40	Total prosthetic replacement of knee joint using cement
W41	Total prosthetic replacement of knee joint not using cement
W42	Other total prosthetic replacement of knee joint
W43	Total prosthetic replacement of other joint using cement
W44	Total prosthetic replacement of other joint not using cement
W45	Other total prosthetic replacement of other joint
W46	Prosthetic replacement of head of femur using cement
W47	Prosthetic replacement of head of femur not using cement
W48	Other prosthetic replacement of head of femur
W49	Prosthetic replacement of head of humerus using cement
W50	Prosthetic replacement of head of humerus not using cement
W51	Other prosthetic replacement of head of humerus

© 2022 Thornhill MH et al. *JAMA Network Open.*

eTable 2. Supplementary *ICD-10* Codes to Identify Whether a Causal Organism Was Recorded and the Nature of That Causal Organism

Causal Organism	ICD-10 Codes
(i) Supplementary codes used to identify all LPJI cases where **a causal organism** was recorded	B95 (and all sub codes) – Streptococcus and Staphylococcus (as the cause of conditions classified elsewhere)<br><br>B96 (and all sub codes) – Other specified bacterial agents (as the cause of conditions classified elsewhere)
(ii) Supplementary codes used to identify those LPJI cases where **oral Streptococci** were a possible cause	B95.4 – Other Streptococcus (as the cause of conditions classified elsewhere)<br><br>B95.5 – Unspecified Streptococcus (as the cause of conditions classified elsewhere)
(iii) Supplementary codes used to identify those LPJI cases where **other Streptococci** were a possible cause	B95.0 – Streptococcus grp A (as the cause of conditions classified elsewhere)<br><br>B95.1 – Streptococcus grp B (as the cause of conditions classified elsewhere)<br><br>B95.2 – Streptococcus grp D (as the cause of conditions classified elsewhere)<br><br>B95.3 – Streptococcus pneumoniae (as the cause of conditions classified elsewhere)
(iv) Supplementary codes used to identify those LPJI cases where **Staphylococci** were a possible cause	B95.6 – Staphylococcus aureus (as the cause of conditions classified elsewhere)<br><br>B95.7 – Other staphylococcus (as the cause of conditions classified elsewhere)<br><br>B95.8 – Unspecified staphylococcus (as the cause of conditions classified elsewhere)

© 2022 Thornhill MH et al. *JAMA Network Open.*

eFigure 1. Sensitivity Analysis Using 3-Month, 4-Month and 5-Month Exposure Windows to IDP Before LPJI Admission (Case-Periods) and Different Control Periods in the Case-Crossover Analyses

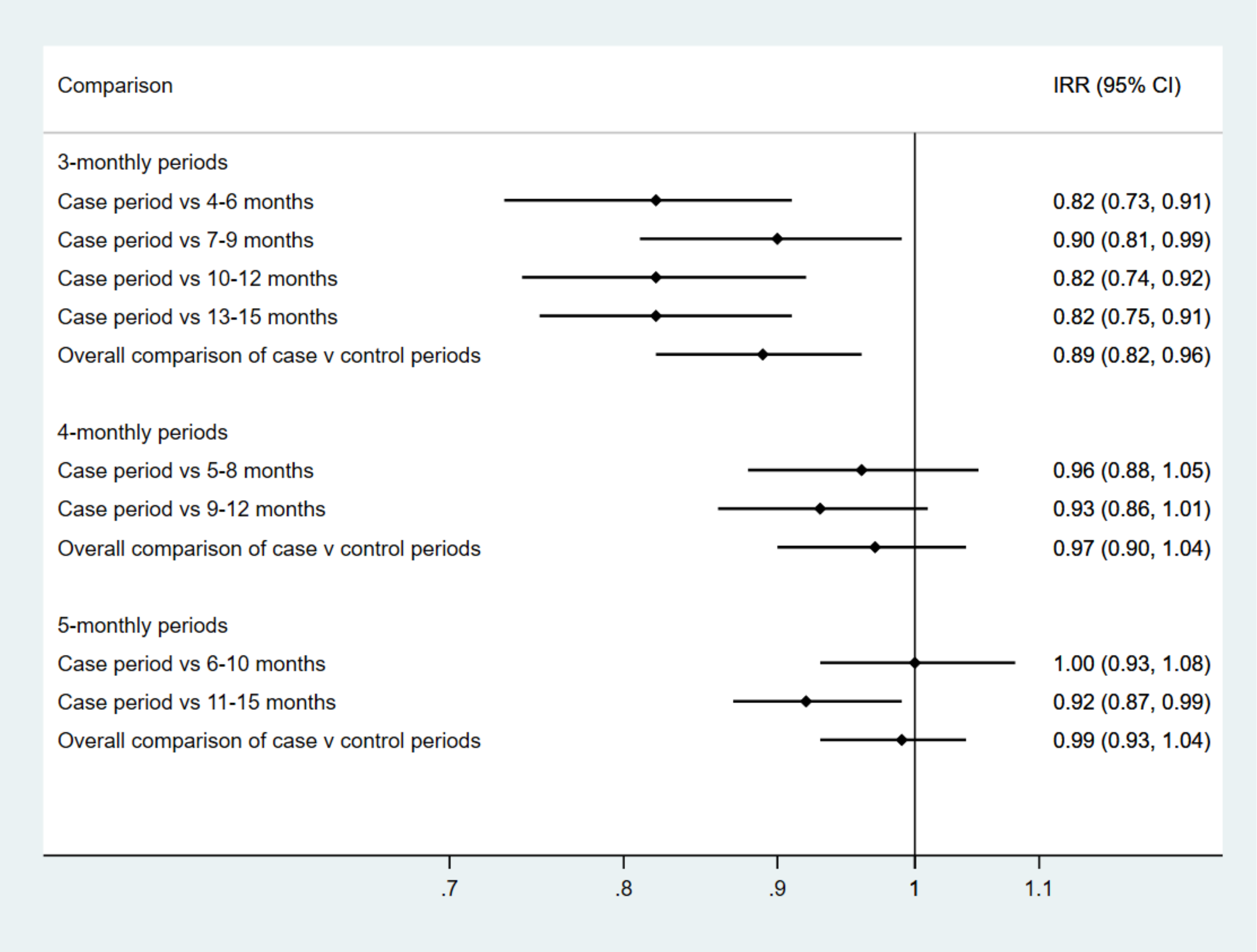

Notes: IDP = Invasive dental procedure, IRR = Incidence Rate Ratio, LPJI = Late prosthetic joint infection.

© 2022 Thornhill MH et al. *JAMA Network Open.*

eFigure 2. Monthly Incidence of Different Dental Procedures Before Hip LPJI Admissions

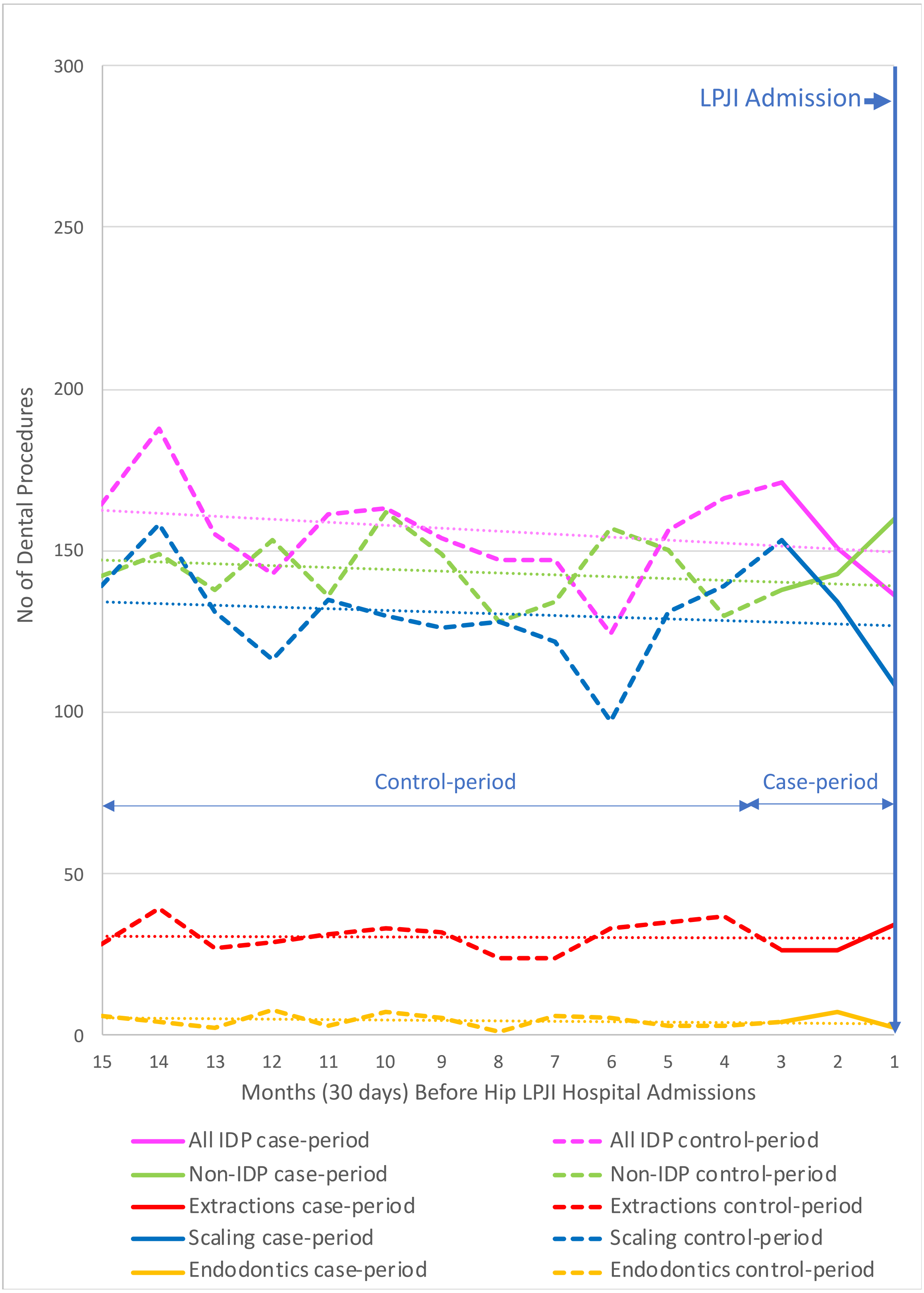

Notes: Solid lines show the monthly incidence of dental procedures during the 3-month case period immediately before IE-admission. Dashed lines show monthly incidence of procedures during the 12-month control period (months 4-15). Dotted line shows the control period trend (extended into the case-period for comparison). LPJI = Late prosthetic joint infection, IDP = Invasive dental procedure.

© 2022 Thornhill MH et al. *JAMA Network Open.*

eFigure 3. Monthly Incidence of Different Dental Procedures Before Knee LPJI Admissions

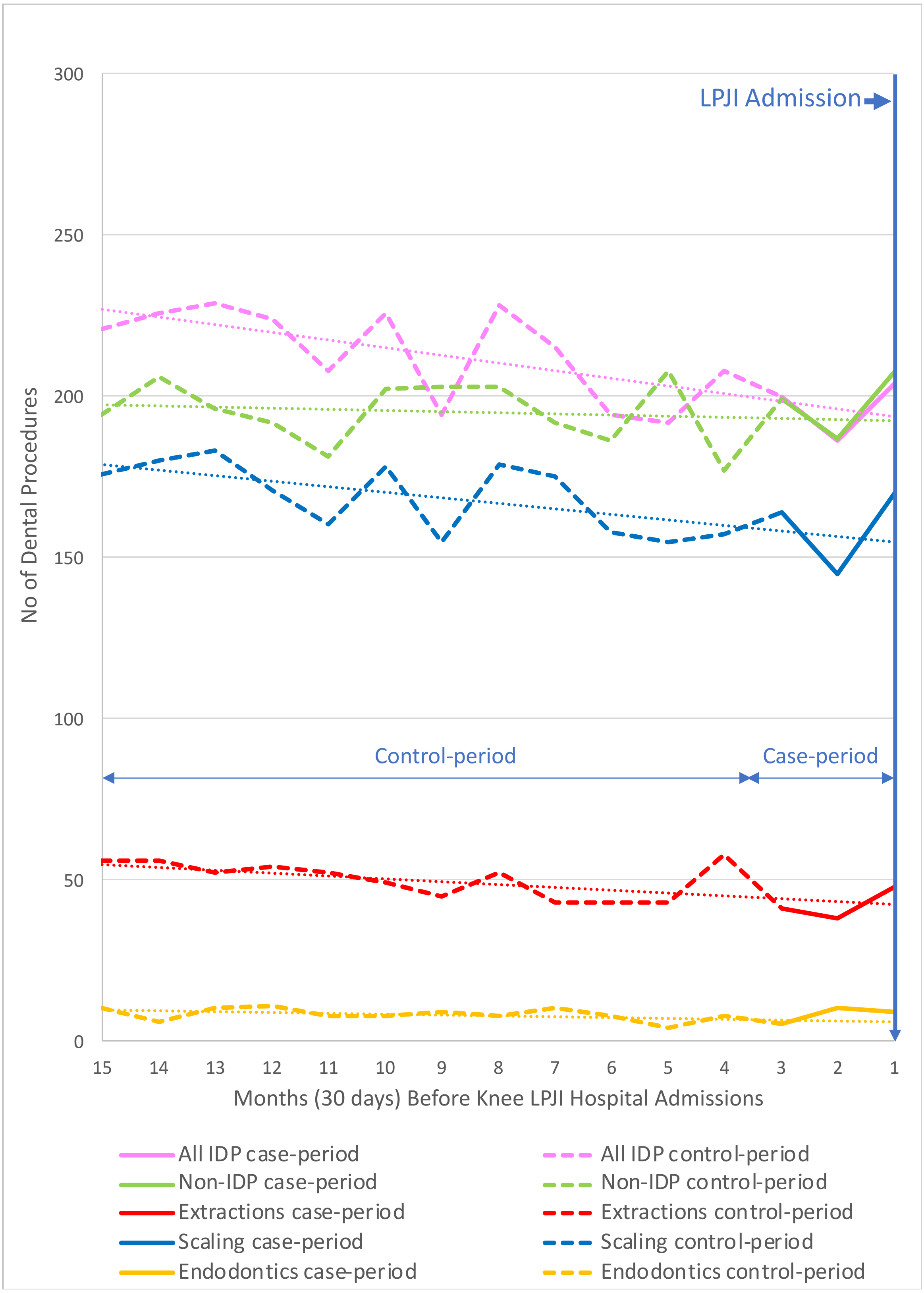

Notes: Solid lines show the monthly incidence of dental procedures during the 3-month case period immediately before IE-admission. Dashed lines show monthly incidence of procedures during the 12-month control period (months 4-15). Dotted line shows the control period trend (extended into the case-period for comparison). LPJI = Late prosthetic joint infection, IDP = Invasive dental procedure.

© 2022 Thornhill MH et al. *JAMA Network Open.*

eFigure 4. Monthly Incidence of Different Dental Procedures Before Other LPJI Admissions

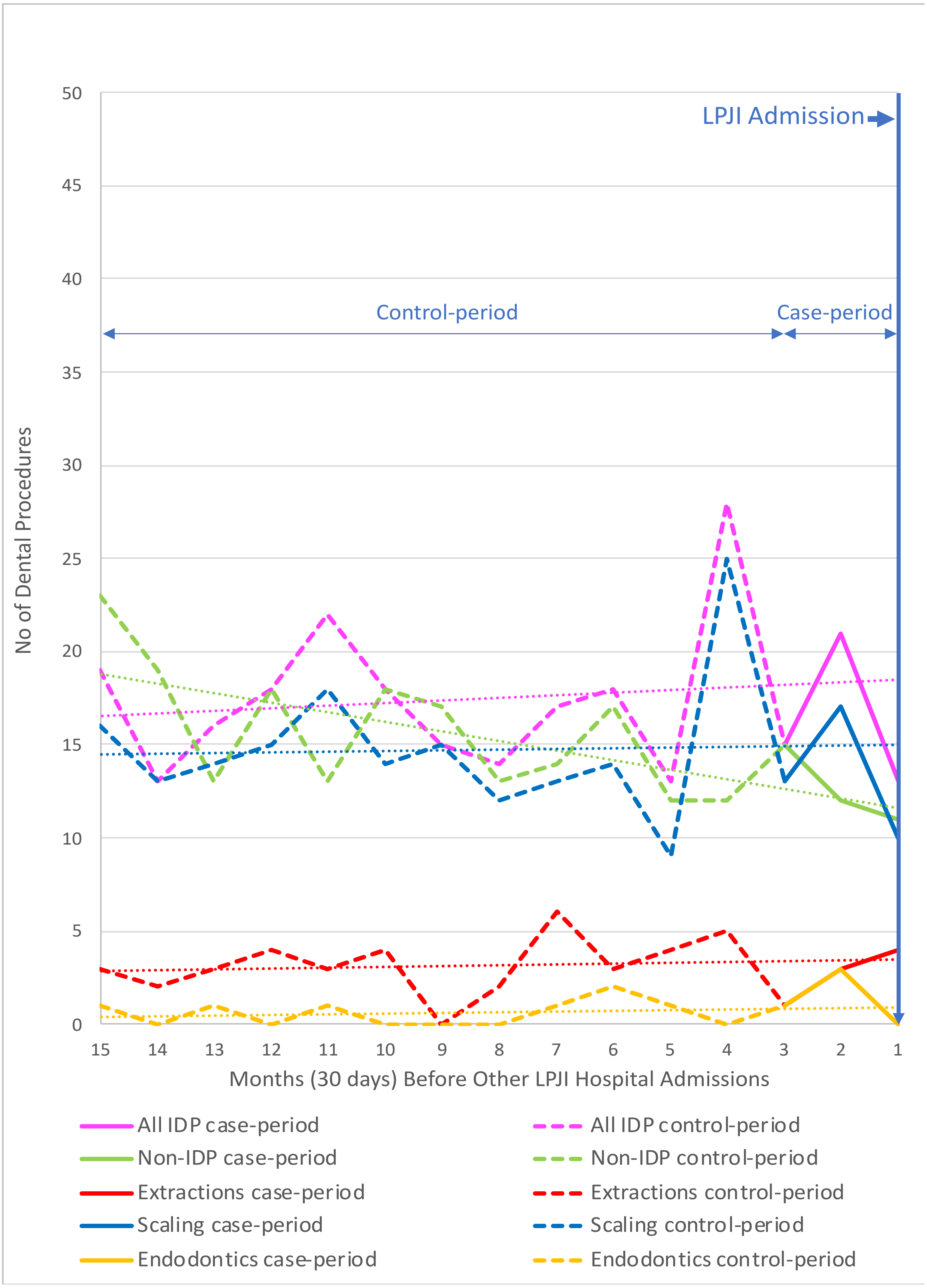

Notes: Solid lines show the monthly incidence of dental procedures during the 3-month case period immediately before IE-admission. Dashed lines show monthly incidence of procedures during the 12-month control period (months 4-15). Dotted line shows the control period trend (extended into the case-period for comparison). LPJI = Late prosthetic joint infection, IDP = Invasive dental procedure.

© 2022 Thornhill MH et al. *JAMA Network Open.*

eFigure 5. Monthly Incidence of Different Dental Procedures Before Unknown LPJI Admissions

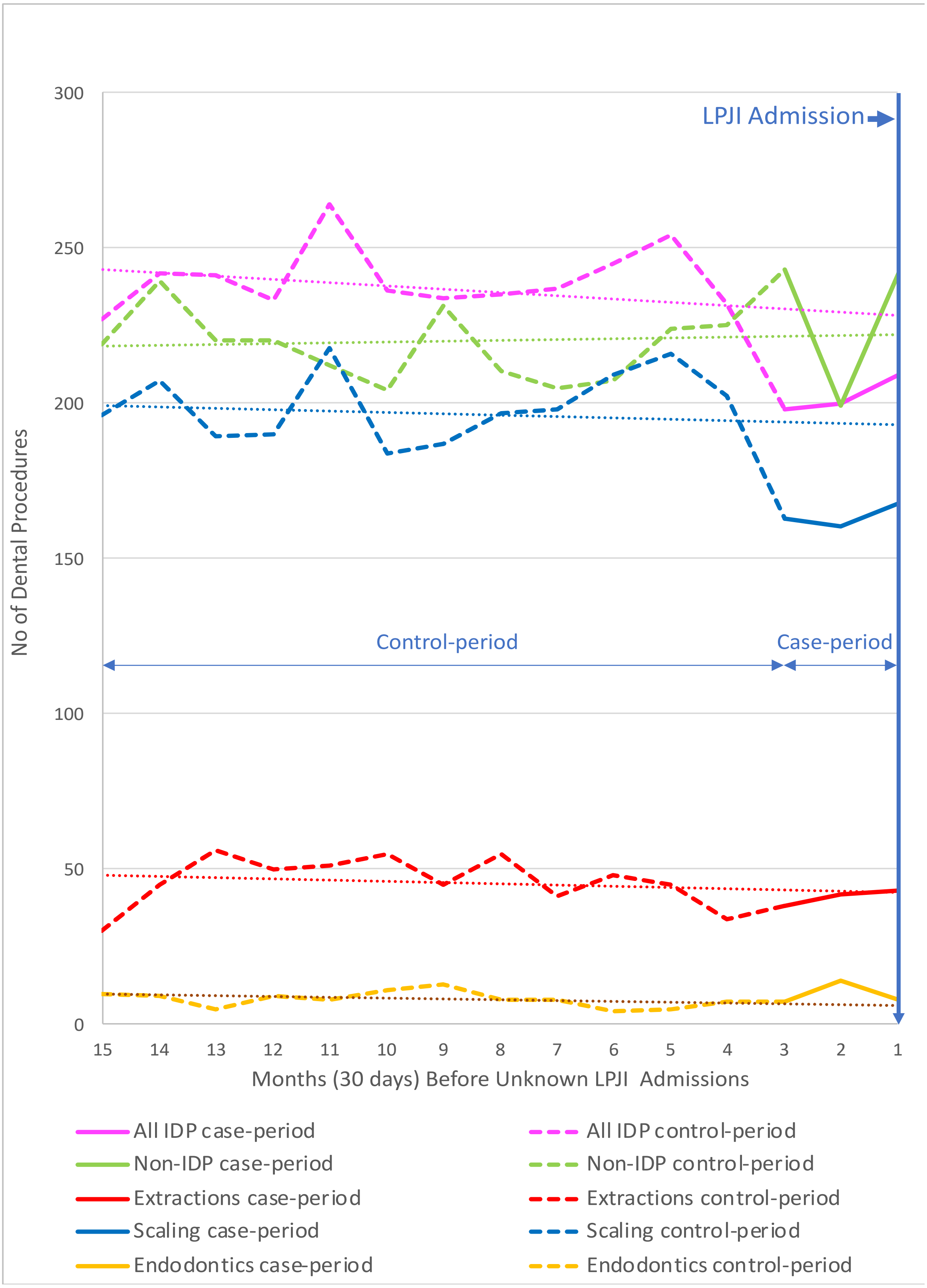

Notes: Solid lines show the monthly incidence of dental procedures during the 3-month case period immediately before IE-admission. Dashed lines show monthly incidence of procedures during the 12-month control period (months 4-15). Dotted line shows the control period trend (extended into the case-period for comparison). LPJI = Late prosthetic joint infection, IDP = Invasive dental procedure.

© 2022 Thornhill MH et al. *JAMA Network Open.*
